# Supplementary material for: Mechanisms governing the pioneering and redistribution capabilities of the non-classical pioneer PU.1
Source: Nat Commun. 2020 Jan 21;11:402. doi: 10.1038/s41467-019-13960-2 (PMC6972792; doi:10.1038/s41467-019-13960-2)
Supplement: Supplementary file 7 — Source data [file 41467_2019_13960_MOESM7_ESM.zip › Source_Data/FigureS2/FigureS2B_MotifScanOutput/KmeansCluster4/homerResults/motif7.similar.html]

motif7

## Information for motif7

C
A
T
G
T
G
C
A
C
G
T
A
T
C
A
G
C
T
G
A
A
C
T
G
T
C
A
G
T
G
C
A
G
C
T
A
T
C
A
G
T
C
G
A
C
A
T
G
A
C
T
G
G
T
C
A
  
Reverse Opposite:  

C
A
G
T
T
G
A
C
G
T
A
C
A
G
C
T
A
G
T
C
C
G
A
T
A
C
G
T
A
G
T
C
T
G
A
C
G
A
C
T
A
G
T
C
C
G
A
T
A
C
G
T
G
T
A
C
  

|  |  |
| --- | --- |
| p-value: | 1e-176 |
| log p-value: | -4.059e+02 |
| Information Content per bp: | 1.606 |
| Number of Target Sequences with motif | 684.0 |
| Percentage of Target Sequences with motif | 15.15% |
| Number of Background Sequences with motif | 1557.5 |
| Percentage of Background Sequences with motif | 3.73% |
| Average Position of motif in Targets | 93.5 +/- 52.6bp |
| Average Position of motif in Background | 104.8 +/- 76.4bp |
| Strand Bias (log2 ratio + to - strand density) | 0.2 |
| Multiplicity (# of sites on avg that occur together) | 1.01 |
| Motif File: | file (matrix) reverse opposite |

### Similar de novo motifs found

|  |  |  |  |  |  |  |  |
| --- | --- | --- | --- | --- | --- | --- | --- |
| Rank | Match Score | Redundant Motif | P-value | log P-value | % of Targets | % of Background | Motif file |
| 1 | 0.773 | G T C A C T G A C T A G A C T G A T C G A C T G C G T A C G T A A T C G C T G A C T A G C T A G G C T A | 1e-57 | -132.969663 | 3.61% | 0.60% | motif file (matrix) |
| 2 | 0.751 | C T A G T C A G T C G A C T G A T C A G C T G A C A T G T C A G C T G A C A T G T C A G C T G A C T A G C T A G | 1e-48 | -111.815193 | 3.46% | 0.67% | motif file (matrix) |
| 3 | 0.793 | A C T G G T C A G T C A A C T G A C T G C T A G C A T G C G T A C T G A T A C G G T C A A C T G C T A G | 1e-29 | -68.650344 | 1.62% | 0.22% | motif file (matrix) |
| 4 | 0.758 | A C T G C G T A C G T A A T C G G C T A A C G T C A T G C G T A A C T G T A C G C G T A A C T G A C T G G C T A | 1e-27 | -64.418915 | 1.71% | 0.28% | motif file (matrix) |
